# Abducted children and youth in Lord’s Resistance Army in Northeastern Democratic Republic of the Congo (DRC): mechanisms of indoctrination and control

**DOI:** 10.1186/s13031-016-0078-5

**Published:** 2016-05-18

**Authors:** Jocelyn TD Kelly, Lindsay Branham, Michele R. Decker

**Affiliations:** Women in War Program, Harvard Humanitarian Initiative, Harvard University, 14 Story St, Cambridge, MA 02138 USA; Discover The Journey, 18 Bridge St Suite 4D, Brooklyn, NY 11201 USA; Department of Population, Family & Reproductive Health, 615 N. Wolfe Street, E4142, Baltimore, MD 21205 USA; Women’s Health & Rights Program, Center for Public Health & Human Rights, Johns Hopkins Bloomberg School of Public Health, 615 N. Wolfe Street, E4142, Baltimore, MD 21205 USA

**Keywords:** Lord’s Resistance Army, Child soldiers, Democratic Republic of the Congo, Children in conflict

## Abstract

**Background:**

Globally, an estimated 300,000 children under the age of 18 participate in combat situations; those in armed groups in particular suffer prolonged exposure to psychological and physical abuse. The Lord’s Resistance Army (LRA) is a rebel movement known for its widespread conscription of children; yet little is known about this process once the group moved beyond northern Uganda. In this paper, we describe the processes related to abduction and indoctrination of youth by the LRA in northeastern Democratic Republic of the Congo ( DRC).

**Methods:**

In-depth interviews were conducted with formerly abducted children, their family members, community leaders, and service providers (total *n* = 34) in four communities in LRA-affected areas of northeastern DRC. Inductive coding of transcripts was undertaken to identify salient themes.

**Results:**

Informants articulated a range of practices by the LRA to exert high levels of control over new recruits, including strict social isolation from recent abductees; control of communication; promoting new identity formation; and compelling children to act out strictly defined gendered roles. Witchcraft and secrecy are used to intimidate recruits and to magnify perception of the group’s power. These methods promote de-identification with one’s civilian and family life; and eventually the assimilation of a new language and identity.

**Conclusion:**

Indoctrination of newly abducted children into the LRA occurs via a complex system of control. This study provides one of the first detailed explorations of social and psychological mechanisms through which this is achieved, and focuses particularly on the gendered differences in the indoctrination process. Results support past findings that the LRA is a strategic and well-organized organization in its approach to enlisting child soldiers. Understanding some of the ways in which the LRA controls its recruits and the psychological impact of indoctrination enables reintegration programs to more effectively address these issues and serve the complex needs of formerly abducted children.

## Background

An estimated 300,000 children under the age of 18 actively take part in combat, or serve as support personnel, for armed groups in more than 30 conflicts worldwide [1]. Children may be forcibly recruited, abducted or coerced into joining non-state armed groups [2]. Once enrolled, these children face prolonged exposure to violence, including but not limited to being forced to kill or harm others, and repeated personal victimization, including sexual violence [3]. Children are considered even more vulnerable than adults to detrimental effects of these experiences, which occur during their formative years [4] and are robbed of many of the standard opportunities for physical, emotional, and intellectual development.

The Lord’s Resistance Army (LRA) is a rebel movement known for its widespread conscription of children and history of perpetrating human rights abuses. Originating in northern Uganda in the late 1980s, the group is responsible for the abduction of at least 20,000 children and the displacement of over 1.9 million people in Northern Uganda [5]. Seminal work on the LRA’s operations in Northern Uganda clarified the LRA’s methods of forced recruitment and psychological control. The LRA is described as preferentially abducting children and adolescents due to the relative ease of indoctrinating youth, and their tendency to stay longer in the group, and their tendency to exhibit more loyalty [6]. Methods of indoctrination included the promise of material gains, such as rewards when the LRA toppled the Ugandan government; the use of misinformation (limiting access to peace-oriented radio messages); the use of violence and the threat of punishment; moving new abductees far from home to disorient them; and inculcating new members to a shared magical belief system. Self-reported loyalty to the LRA was high among abductees, and increased with time in the group [6].

In 2005, however, the LRA was forced to expand its territory and seek operating bases in neighboring countries in response to military pressure from the Ugandan government in an attempt to regain stability in the northern region of the country. The LRA expanded across the western border of Uganda to the Democratic Republic of the Congo (DRC) as well as to the north in South Sudan, and Central African Republic (CAR). Expansion into DRC and other countries presents a unique challenge to the rebel group, which has based its identity and much of its philosophy in pride around an Acholi tribal identity. Outside of Uganda, the LRA’s abductees include those with different backgrounds, language and culture, adding a layer of complexity to the forced indoctrination process as their historical leveraging of the Acholi tribal identity as a reason to fight with the group is no longer relevant [7]. Yet unclear is the means of indoctrination in contexts outside of Uganda that do not share a common language, tribe, history or culture. It is critical to understand how the LRA may have changed or adapted its behaviors in response to the diffusion of the group across the central Africa region. Considering potential gender differences in these experiences is also critical. In a report to the Security Council, the United Nations estimated that between July 2009 and February 2012, the LRA had abducted 591 children, with a roughly even split between girls and boys in the DRC, South Sudan and CAR [8].

The theoretical grounding for understanding indoctrination practices is sparse. However, in an examination of identity construction experienced by returning former child soldiers in Uganda, Veale and Stavrou [9] note the extent to which identity is constructed through relationships between inductees to a new culture, and the long term practitioners who take on a role of modeling accepted behaviours. This learning process is enacted through daily activities and through social relations with the older, “expert” actors in a group [9].

The stages of this kind of learning and indoctrination process were examined in detail in an exploration of how youth become “insiders” in different types of violent communities. In the article “Becoming a Committed Insider,” Hundeide [10] compares case studies of children joining neo-Nazi groups in Europe with the experiences of child soldiers in Angola and Sierra Leone. While emphasizing that there may be different motivations and pathways to transitioning from “outsider” to “insider” in a violent group, she outlines the following steps, which may occur in tandem or in different order as youth identify with a violent sub-culture: separation and anxiety; identification with the aggressor; terror and the breaking of pre-existing emotional bonds; undermining earlier morals; isolation and indoctrination of new values; demonization of the enemy or “outsider”; and finally, becoming insiders into the new group.

Terror and the breaking of previous ties may take the form of committing the most extreme forms of violence against one’s own community. This can serve to then undermine earlier morals as well as make new recruits feel isolated and bound to a new set of values. Betancourt et al. found that two forms of violence – killing and rape – were particularly damaging to long-term mental health outcomes for children formerly associated with armed groups [11]. Further supporting this finding is evidence from a study with former child soldiers in Uganda, which found that being forced to kill, and witnessing killing were reportedly the two worst kinds of experiences they underwent [12]. The findings from Betancourt et al and Ertl provide evidence that certain extreme forms of violence do indeed create a lasting break with accepted norms, and may carry consequences far after an individual leaves an armed group.

This study explores how different practices associated with indoctrination – particularly isolation, assignment to a new family structure and forced violence – may serve as the most effective way to make new recruits feel that they have been irrevocably dissociated from their civilian identities, and that they would no longer be accepted in their own communities.

The growing recognition of the role youth play in conflict [13–16], and recognition of the trauma they are exposed to [3, 17–21] has brought increasing efforts to improve reintegration programming. However, service providers often struggle to address the needs of children associated with armed groups. Systematically understanding the experiences of abductees in this context, and creating programs that specifically address these experiences, could improve programming in this area. Examining the experiences of children indoctrinated into the LRA, and particularly the psychological dimensions of their experience, can inform context-appropriate services to best address their needs upon return to civilian communities. This research begins to fill this gap by exploring the LRA’s mechanisms of indoctrination after its expansion into neighboring nations. We describe LRA indoctrination of abducted children and young adults in northeastern DRC, as reported by former abductees, their family members, and other key informants in LRA-affected communities.

## Methods

The study was conducted in the Haut Uele district within the Orientale Province of northeastern DRC, a rural region bordered by South Sudan to the north, Central African Republic to the west and Uganda to the east. The predominant tribe is Zande, who traditionally engage in subsistence agriculture, while the minority nomadic pastoralist tribe, the Mbororo, move across the region seasonally. In 2011, during the time that this research was carried out, the United Nations Office for the Coordination of Humanitarian Affairs (OCHA) reported that 443,702 people had been displaced by the LRA in the Orientale Province since 2003, with roughly 60 % of this displacement (257,265 individuals) occurring in Haute Uele. The towns of Dungu and Faradje were home to over three-quarters of those displaced in Haute Uele [22].

Semi-structured qualitative interviews were undertaken with informants in four communities in LRA-affected areas of northeastern DRC: Duru, Faradje, Limai and Dungu. These communities were chosen because they were among the most affected by the LRA, according to informal interviews with local leaders, non-governmental organization (NGO) staff and other key-informants. At each project site, the research team, consisting of a local religious leader, a local translator and two U.S. researchers, first met with local community leadership to describe the planned project, answer questions, and obtain project approval. In the Dungu site, interviews were also conducted at a nearby displacement camp with roughly 4000 residents, to examine the needs of those displaced throughout Haut Uele.

### Sample and recruitment

Recruitment occurred via snowball sampling. The team began with multilateral organizations and international NGOs working with communities affected by the LRA in the sample area, and obtained referrals to additional key actors who had experience working with LRA-affected communities and individuals: these included international NGOs, local religious organizations and community-based organizations. The team ceased seeking out new informants when they were no longer receiving new referrals. Since family, community members and service providers are deeply affected by LRA violence, and are closely involved in the reintegration of formerly abducted youth; these individuals were sought for interviews to provide their perspective on the conscription of children and the process of reintegration. Data was de-identified except for demographic characteristics (age, role in the community). The final sample (*n* = 34) included formerly abducted children (*n* = 7), their family members (*n* = 3), community leaders (*n* = 11), service providers (medical and educational staff and NGO workers (*n* = 13).

### Data collection and analysis

The semi-structured interview guide was designed based on informational interviews with NGO staff and community members in affected areas, and refined after pilot interviews in Dungu. Interviews were conducted in Zande (the local tribal language), or French, based on participant preference. Following a semi-structured guide, participants were asked to describe their understanding of LRA abduction practices, whether experienced personally or understood through the context of service provision, the impact of the LRA, feelings of vulnerability, any gender differences in experiences with the LRA, reintegration of children and adults formerly abducted by the LRA into communities, and community coping and resilience strategies towards the ongoing insecurity. Probes were used to elicit details. A translator and one to two researchers were present at each interview. Interviews were transcribed verbatim during the interviews. These notes were combined for completeness, and were reviewed for accuracy and translated into English language for analysis.

Procedures aligned with ethical guidance for research with children in war-affected populations [23, 24]. Consent procedures emphasized the voluntary nature of participation. Local interviewers were trained in qualitative research techniques and were service providers with local organizations that had experience working with LRA-affected populations. In accordance with best practices, interviewers were trained to monitor participants for possible distress, and emphasized that respondents could refuse any question. All respondents were interviewed in private locations. Interviewers were trained using training materials developed from materials provided by a university IRB. The training materials covered core ethical concepts including: privacy and confidentiality, proportionality, risk, consent, beneficence. All IRB materials were translated into French and were printed and reviewed in depth with local research staff during a 2-day training. Referrals for mental health services were provided to all participants following the interview. This work was undertaken as part of an NGO needs assessment, and as such was deemed exempt by the Harvard School of Public Health Institutional Review Board. Community leaders and service provision organizations reviewed and provided input on procedures, and gave permission to engage with community members.

Inductive coding was undertaken through close reading of all of the transcripts to identify salient themes. Two research team members generated themes independently; those that were agreed upon by both researchers were defined as codes. Themes were refined through an iterative process during the coding of the transcripts to capture emerging themes and subthemes. Through this process, key unifying themes and relationships among these themes were identified. Coding was undertaken by one team member and cross-checked by another. Consistently appearing themes within categories were captured as subcategories. Data was coded in a database in Microsoft Excel (Microsoft Excel 2011; Version 14.0.0).

## Results

Themes are presented in roughly the same order as the process of indoctrination: abduction; steps undertaken by the LRA to establish control through isolation and control of communication; the use of public punishment to intimidate and deter escape; assignment to a unit within the hierarchy; and, finally, markers of the transition into becoming a full LRA soldier. The last theme explores the use of witchcraft to intimidate recruits and to magnify perception of the group’s power throughout the indoctrination process. Table 1 summarizes the mechanisms of control and Table 2 illustrates the gendered similarities and differences throughout the process of indoctrination.

**Table 1:** Mechanisms of control

Isolation from others who share a common language and culture	“If they see you talk with one person frequently, they think you want to run away and they will beat you up.”
	“When they kidnap you, you just move without any rest and you can’t talk with each other, especially those who were abducted. And if you do, they will say you are planning to run away and they will beat you.”
Control of Language	“If you are a boy, you stay with them 1 year, you learn the language and become a soldier.”
	“When I was kidnapped since I didn’t know their language – they were forcing us and beating us to learn their language”
Public Violence and Forced Complicity	“They found 2 boys from the Zande tribe who tried to escape and they pulled other Zande boys from the group and forced them to beat those who tried to escape with sticks until they died.”
	“If one child tries to escape, they catch him, put him in the middle of a group of children and make the children kill the child with a piece of wood and say if you try to escape we will kill you like that.”
Assignment to family structure with gendered roles	“…normally when they train other kids to be LRA they put them under someone’s responsibility.”
	“My job was basically to carry kids, cooking and carrying things when we were moving… [The man they gave me to] had other wives.” (female abductee)
	“Boys in the LRA are transporters, then they give the gris gris and they become LRA.” (male abductee)
Witchcraft	“When children come back [from the LRA], they are troubled, mean. They don’t play with others. They have *gris gris* that torments them.”
	“ The behavior [of the recipients] changes when they get witchcraft…”

**Table 2:** Phases of indoctrination

Girls	Silence and isolation	Assignment to a family structure, learn Acholi language	Help with domestic tasks, become “bush wives if they are deemed old enough	
Boys	Silence and isolation	Assignment to a family structure, learn Acholi language	Given increasingly difficult tasks to test their loyalty	Trained to become a soldier where they can earn the right to carry a gun and be given a “wife”

### Abduction

In this study area, respondents universally emphasized the violent and coercive nature of abduction by the LRA. Kidnappings often occurred as part of a larger attack on a village. Interviewees described a preference by the LRA to take children and youth, rather than adults, because children are easier to control. They also noted that both boys and girls were vulnerable to being taken.

*“Children are abducted by force into the LRA. They don't have a choice to accept or not accept. If you accept to go, you live. If you don't accept they kill you.” -*Religious Leader

*“Children never volunteer, they are kidnapped on the road, on the field, even in the village, that are trained in a military way and become the LRA.”* - Mother of a formerly abducted child

Respondents expressed how the LRA preferred to abduct children, who could more easily be indoctrinated to the group’s practices, compared to adults.

*“For men, it is terrible because when LRA finds you, you are killed directly, no discussion. Children will be kidnapped.”* – Leader of a displacement camp

*“We started looting people and killing people, abducting children from the village and bringing them to the [LRA] camp. Those who were older, we would kill them.”* – 17 year-old male abductee

There was consensus among the respondents that boys and girls were equally vulnerable to being abducted by the LRA. However, once within the group, gender roles were rigidly defined. *“All are vulnerable to abduction - girls and boys.”* - Chief of Duru village

*“Boys and girls are equally vulnerable. Boys are fighters, porters. Girls are wives and get pregnant.”* – 18 year-old female abductee

However, once within the group, gender roles were rigidly defined and strictly enforced through a number of mechanisms. One of the first and most striking aspects of life in the LRA is the strict control of roles and behaviors that those in power exercise over others.

### Isolation and control of communication

Male and female respondents described the forced indoctrination process in similar terms. After being kidnapped, many new abductees reported being forced to march for hours and sometimes days into the forest. During this time forward, abductees were not allowed to speak in their native languages or communicate with others. Former abductees said that these measures were intended to prevent children from bonding with each other and from forming escape plans.

*“[We] couldn’t talk to other abducted kids. We just sat and slept the three days I was with the LRA.”* - 17 year-old male abductee

Congolese children describe spending days hiking and transporting looted goods, or sitting in groups to rest in total silence.

*“If they see you talk with one person frequently, they think you want to run away and they will beat you up.”* Another girl reinforced this, saying, *“When they kidnap you, you just move without any rest and you can’t talk with each other, especially those who were abducted. And if you do, they will say you are planning to run away and they will beat you.”-* 16 year-old female abductee

Isolation was focused on ensuring that fellow abductees did not interact or bond with each other. However, interaction with, and assimilation to, the language and customs of long-term combatants was cultivated, ensuring that one’s ties to a civilian identity were broken while new ties to an LRA identity were reinforced.

### Forced language acquisition & language as indication of identity

Participants described a number of ways that the LRA has adapted to the challenge of working in a country that does not share a language or common identity with the combatants. Respondents emphasize that recruits have different backgrounds, language and culture than the majority of the LRA^1^- adding a layer of complexity to the process of forced indoctrination. This is a challenge the group has addressed with brutal efficiency, severely beating any new members who talk amongst themselves as well as those who use a language other than Acholi. This compels abductees to quickly learn the language of the LRA in order to communicate.

*“We couldn’t talk to other abducted kids. We just sat and slept…”*- 17-year-old male abductee (from Faradje)

This is most pronounced in those cases where children are assigned new names –effectively stripping them of their identities and rechristening them as members of the LRA.

*“When I was kidnapped since I didn’t know their language – they were forcing us and beating us to learn their language*… *When they abduct you they ask your name and then if it’s too difficult they give you a new name.” –* 17-year-old male abductee (from Dungu)

Community members as well as former abductees closely linked learning the Acholi language with the process of taking on a LRA identity. While not all community members associated the Acholi language with LRA soldiers, there was wide recognition of the fact that abductees who spent enough time in the group learned this language.

“*If you are a boy, you stay with them one year; you learn the language and become a soldier.”* – 18 year-old female abductee

*“Once we hear Acholi we think he is the enemy even if they are not in uniform.”* - Leader displacement camp

Service providers working with abductees stated that children often avoided speaking this language once they returned to their communities because it served as a marker of being an outsider and emphasized their previous identity with the LRA. Abductees, family members and service providers drew the connection between speaking Acholi and being identified with the LRA.

*“My daughter is behaving and is calm. She hasn’t changed but I know a boy who spent much time in the bush and he is angry, is violent and only knows Acholi.”*- Father of female abductee

### Public punishment to intimidate

Public violence was described as a way to ensure that abducted children were aware of the price of disobedience. Killing and beatings were used to discourage escape in particular. Participants explained how LRA command would bring the transgressor back to the group and kill him or her in front of the other abductees in order to send a public message.

*“If one child tries to escape, they catch him, put him in the middle of a group of children and make the children kill the child with a piece of wood and say if you try to escape we will kill you like that.” -* Leader of the refugee camp

*“If someone tried to escape they would go, follow him, bring him [back] and kill him,”* - 17-year-old male abductee

These practices were also used to purposefully deconstruct social ties by forcing children to perpetrate violence against someone similar who shared their tribe or ethnicity.

*“…if a boy or girl tries to escape, they will be brought back and put into a circle and all the boys and girls will kill those children.”* – Female NGO worker

*“It is difficult to leave the LRA because they scare children and say if you try to escape, they will chase you and kill you. They found two boys from the Zande tribe who tried to escape and they pulled other Zande boys from the group and forced them to beat those who tried to escape with sticks until they died.”* – 18 year-old female abductee

The use of public violence not only served to intimidate children into staying in the group and obeying orders; by forcing abductees to kill or beat their peers, commanding officers used displays of violence to break social ties and make children feel complicit in the brutality.

### Transience and instability

Life in the LRA was described as, above all, highly transient. Long periods of living in the forest were punctuated by raids on villages where abducted Congolese children witnessed and participated in violence, which also served to break bonds with their civilian identities. This existence, characterized by constant movement and punctuated by violence, meant that children had no sense of stability or community beyond those they are traveling with.

*“What I was seeing was that, when there is no food, we would go to houses and look for food and when they go to the house they kill the owner of the house. We stay in the bush until food is finished and when it’s finished we go again to loot for food.”* – 16 year-old female abductee

Keeping abductees on the move ensured their survival became dependent on the LRA commanders. Forced to forage for food in the forest and to undergo spontaneous fasting periods until the next raid, basic survival became linked to obedience to the command structure and to staying with the group.

### Assignment to a “family” structure

Respondents described how each raid had the possibility of bringing new recruits who were needed to help carry the supplies from the village that had just been attacked. This influx of new abductees meant that the LRA had to find a more sustainable way beyond silence and isolation to condition new members. This was done through assigning abductees to a trusted older combatant, often from Uganda, who already had an existing “family structure” consisting of one or multiple women who were forced to act as wives as well as their children. Control of new recruits was thus “contracted out” to smaller units where more effective supervision was possible.

*“There was somebody – the one who abducted me also would give me some food – I was the one carrying his food and his wife’s goods… He had a rank – the ranking is complicated in LRA – normally when they train other kids to be LRA they put them under someone’s responsibility. So he was responsible for those kids and other LRA members.”* – 17 year-old male abductee

In this “family” unit, abductees are the lowest ranking members and are subject to beating and other abuse. The children’s behavior in these units is highly conscribed. Girls are controlled not only by the combatant they are assigned to, but also by other wives and family members in that social unit.

*“When they abduct kids, especially girls, they distribute them to the commanders – they say, 'you, this is your husband, this is your husband.' Those who are not mature will give to other commanders and say, 'you keep this kid until they are old enough to be your wife'… My job was basically to carry kids, cooking and carrying things when we were moving… [The man they gave me to] had other wives. They were Ugandan. They were not nice to me – the Ugandan wives.”* – 16 year-old female abductee

For boys, this process reaches its final stages when they are “graduated” to becoming combatants. This process involves staying with the LRA for a significant amount of time. Once boys have been with the group for a year or more, participants described how boys were given increasingly difficult tasks to test their loyalty, for instance being sent on an errand to a distant place. His return was seen as an indication of his loyalty to the group.

*“Boys will be taken, if they don’t want to go back home they will be trained as fighters, they will ask a boy questions to see if he is trustworthy, then they will send him to fetch water, if he keeps coming back, they will train him.”* – 18-year-old female abductee

The process of becoming a soldier can then begin, and is characterized primarily by the use of witchcraft and secretive rituals. In the final phase, a boy is given a gun and eventually a wife so he can start his own “family” unit.

### Witchcraft

Respondents describe the use of witchcraft, or *gris gris* as it is often called in DRC, as a defining part of the LRA experience. Although respondents often did not know the purpose of the magical rites they experienced, they placed an emphasis on its importance, and even after escaping from the LRA expressed a fear of its power. Magic was used at several points during the abduction and conscription process; immediately upon abduction, and later as a symbol of transition into becoming “true LRA.” A number of children describe undergoing a magical ritual after first being abducted, often in the form of oil or cutting.

*“The LRA put the* gris gris *on our ankles and knees and on our heads. It was oil in a small bottle. When they abduct children they put this oil on them.” –* 17-year-old male abductee

*“Only when they abducted us I saw that first they apply witchcraft on the knees and on the ankles but I don’t know why they applied it but they applied it. They applied it on me. It was like an oil. Only that first time – it never happened again. I don’t know why.” –* 16-year-old female abductee

*“My son told me they took a razor and cut his right leg, left leg and forehead when he was abducted. This was a form of* gris gris*.”* – Father of a male abductee

While some noted that magic was applied immediately after abduction, it was also common to hear of *gris gris* as a way to signal the transition from an abductee to a true soldier.

*“Mostly the chief of the [LRA] group is in charge of the witchcraft and when someone stays with the LRA for more than one year, they will choose who gets the witchcraft and take them away from the group and apply it on them… The behavior [of the recipients] changes when they get witchcraft – they cooperate amongst themselves they become very aggressive…Boys in the LRA are transporters, then they give the* gris gris *and they become LRA.””* – 17-year-old male abductee

The descriptions of witchcraft seem to be a method for describing the process of indoctrination that some children undergo in the LRA, and shorthand for the extent to which children then become part of the group. A local NGO worker in Limai explained:

*“Normally the LRA makes some kind of witchcraft and puts it on the kids – witchcraft from Uganda – we don’t know how to undo this witchcraft. If it was done here maybe we could undo it but it was done in Uganda so we don’t know how to undo it… The children bring bad things from the bush… once they are brought to the bush with the LRA and given witchcraft training or something; he comes back different and brings bad things from the bush.”* – Male NGO worker in Limai

*“Children [in villages] were mostly killed by LRA and those kids who had witchcraft on them… Only kids with the witchcraft would kill people.” –* 17-year-old male abductee

This was echoed by family members of abductees and the children themselves, who looked at receiving witchcraft as an indication of the mental changes one undergoes in the group.

*“They use drugs and* gris gris *in the LRA to change attitudes but she [my daughter] didn’t take the magic because she was hurt during her capture and so was being healed rather than being indoctrinated.”* – Parent of female abductee

*“It is since the LRA didn’t apply their potion on me that I am ok and my mind is ok.”* – 17 year old male abductee

*“When children come back [from the LRA], they are troubled, mean. They don’t play with others. They have* gris gris *that torments them.”* – Teacher from Limai

## Discussion

This study is among the first to examine the ways that the LRA indoctrinates youth outside of northern Uganda, where new recruits no longer share a common cultural background or language with long-term soldiers. These findings also contribute to the wider literature examining the processes by which children are indoctrinated into violent organizations. The LRA has relied heavily on forced recruitment and abduction of children to fill its ranks, and has adapted systems for breaking ties with civilian life and fostering new identity formation. Understanding the processes through which this occurs, especially as the group expands into areas that share no linguistic or cultural ties with the long-term combatants, provides a rare insight into methods of indoctrination and psychological control for children associated with armed groups. These findings illustrate a highly regulated process of indoctrination in four project sites in northeastern DRC. Participants described a tightly-controlled process of manipulation, including: immediate isolation from their peers and fellow abductees after abduction; forced acquisition of a new language; constant movement and occasional violent raids that serve to dissociate children with civilian life; assignment of new abductees to a “family” structure where they are supervised by a senior combatant and his wife or wives; and finally, the use of witchcraft for intimidation. This paper supports evidence that the LRA is a strategic and well-organized organization, [25–28] despite its depiction in Western media as an irrational band of rebels with arcane religious beliefs [29].

These results also reinforce previous studies which posit similar stages of identity formation. As noted in the introduction, Hundeide outlines potential routes for identifying with a violent sub-culture: separation and anxiety; identification with the aggressor; terror and the breaking of pre-existing emotional bonds; undermining earlier morals; isolation and indoctrination of new values; demonization of the enemy or “outsider”; and finally, becoming insiders into the new group [10]. These findings emphasize how the LRA enforces separation both from the civilian community (through abduction) and from other abductees in the group who share a tribal or ethnic identity (through control of language and by separating recruits into smaller family units). The use of family units and of a magical belief system helps ensure that new recruits are controlled socially and psychologically.

Studies from Sierra Leone [11] and Uganda [12] reinforce the ways that extreme violence, including killing, rape, and being forced to witness killing, are especially harmful for youth in armed groups. It is notable that being forced to perpetrate murder is one of the defining experiences of many children associated with the LRA, and is discussed by the respondents in this study, as well as being noted in previous descriptions of the LRA’s practices in Uganda. The use of violence and punishment, and the movement far from home to prevent escape, was also reported among Uganda recruits [28]. The influence of evil spirits to control children and as a barrier to reintegration is also consistent with findings in the Ugandan context [30].

Current findings extend this literature by identifying additional methods – such as control of communication with those from a similar background and the forced acquisition of language, that appear unique to the LRA’s new context. Some methods seem to have been kept but adapted. Current results suggest magic was used for intimidation, contrasting with past work in Uganda where the magical belief system was described as a shared “cosmology” that both inspired and scared abductees [30]. In the post-Ugandan context, magic has been documented as highly instrumental in the LRA and is used to exert psychological control by: discouraging escape, inciting higher levels of motivation, providing legitimacy to the group and its commander, fostering group cohesion, and intimidating civilian populations [31]. Other scholars have documented how magical beliefs can serve highly specific strategic functions in armed groups in Africa [32–34].

Findings demonstrate the measures taken by the LRA to force recruits coming from outside of Uganda to feel, if not equally loyal, at least equally bound to the group. This appeared to be achieved through re-formulating some of the most basic concepts of social bonding, for example the concept of a “family” unit. Abductees are also systematically prevented from forming relationships with those from their own culture. Children are coerced into keeping silent until they learn the Acholi language and stop using their own. And finally, the constant movement and transience may make it difficult for abductees to create ties with a stable home community upon their return. The results presented here provide qualitative support for the finding from Betanacourt et al that certain forms of violence – killing and rape – are particularly traumatic for children associated with armed groups. These forms of violence, because of their intense social taboo and associated stigma may be among the most effective at preventing children from feeling that they can escape the group and return to a normal life. A recent qualitative study from those associated with the LRA outside of Uganda emphasized the extent to which being forced to commit violence and murder, particularly against those in one’s own family, served as a way to disconnect people psychologically from their civilian past and was interpreted by those abducted as a way to communicate that there is no way to return to their past life [31].

In this study, being forced to kill and experiencing sexual slavery were defining aspects of the indoctrination process, and the forced formation of an LRA-type identity. Additionally, the descriptions of language control, of forced isolation, and of the assignment to family units where long-term combatants could model and enforce “approved” behaviours, speak to highly systematic ways of forcing the formation of a new identity.

Gender differences emerged in how youth were treated and indoctrinated. Girls are assigned as cooks, carriers, and wives of senior LRA combatants. They often face control not only by their combatant “husband” but also by other women who act as wives in this “family” group. In contrast, boys first act as transporters and, with time, gain increasing responsibility as combatants. Violent rituals, as well as tests to prove loyalty, often mark the transition to becoming a combatant. While both boys and girls face control, fear and violence, the character of their experiences may be markedly different. Girls suffer physical and sexual violence within their family units. Boys face physical violence and domination as they are molded into potential fighters. These findings highlight a changing approach to women by the LRA. Female abductees were trained as combatants during the LRA’s activities in Uganda [35, 36]. However, as the group became weakened after its dispersal to other countries, the majority of those trained for combat were boys and young men. Recent interviews with long term LRA combatants describe how currently, women and girls abducted into the group after this time served as “wives” and servants. Only those women who were unable to fulfill these roles due to disability or undesirability were put on the frontlines of fighting [31].

All of these realities have consequences for those individuals seeking to rebuild their lives in civilian communities once they leave the LRA. Upon reintegration into their communities of origin, abductees must re-create social bonds, communicate with family and acquaintances, and regain autonomy over daily activities that were once dictated by others. Recognizing these realities in programming is important. Returnees may experience mental health issues, may be at increased risk of re-recruitment, and may be more vulnerable to exploitation [11, 37, 38]. Former child soldiers associated with the LRA in Uganda report experiencing stigma from host communities, though this may decrease over time [39], and former child soldiers in the conflict in eastern DRC reported significant stigma from host communities, which is expressed through name-calling and social ostracization [40].

Psychosocial interventions should therefore address not only individual healing of former abductees, but focus on helping these individuals once again build communication and trust skills with family members and peers. A randomized control trial conducted in LRA affected areas in DRC for war-affected boys, including former child soldiers, found that cognitive behavioural therapy (CBT) modified to be culturally relevant, proved effective in improving mental health [41]. Another study enrolled 159 war-affected youth in LRA-affected Haut-Uele Province of DRC into a community-participative psychosocial intervention involving trainings with life skills and relaxation. Immediately post-intervention, youth showed significantly lower post-traumatic stress reactions compared to controls and positive effects continued to be evident three months post-intervention [42].

Previous studies from Sierra Leone highlight the fact that disarmament, demobilization, and reintegration (DDR) programs are often deeply inadequate to address the long-term mental health and economic needs of former child soldiers [37], and highlight the unique needs of girls and women associated with armed groups [43]. Qualitative data from former child soldiers and community members participating in DDR processes in eastern DRC suggest a number of promising practices, including promoting community and family involvement at all stages of reintegration programming, and training local service providers to provide counseling to former child soldiers and their families [40]. These findings echo research done in LRA-affected areas in Uganda. There, resources seen as important for reintegration were: spiritual healing and religion; sensitization messaging for stigma reduction; counseling; and joint activities between community members in reintegrating individuals [30, 39, 44–47], with an emphasis on the importance of holistic interventions that specifically take into account beneficiaries’ social and cultural environment. Home visits and counseling messages with a spiritual or religious message have also been found to help address trauma [48, 49]. Holistic interventions that involve positive social interactions between community members, care givers and abductees have been recommended in other LRA-affected contexts [47], as have short-term trauma-focused treatment for those most highly affected by violence [12].

### Limitations

Findings should be considered in light of several limitations. The generalizability of findings beyond the geographic areas included in our study is unclear. Security and logistical constraints rendered some regions, including the highly impacted Bas Uele region, inaccessible. Despite efforts to include a diversity of experiences, our sample is limited to those that were most accessible. We were not able to include youth still part of the LRA; they may have had qualitatively different indoctrination experiences. Despite efforts to ensure accuracy in transcription and translation, including clarifying concepts during interviews and debriefing after interviews to confirm accuracy, some information may have been lost.

## Conclusion

The LRA exercises extensive behavioral and psychological control over its new recruits in northeastern DRC. This study provides one of the first detailed explorations of the social and psychological mechanisms through which this is achieved following expansion of this group’s activities beyond Uganda. Understanding abductees’ experiences is critical to guide reintegration programs for youth, such as DDR programs. Findings emphasize the need to consider indoctrination processes specific to the LRA experience when planning reintegration activities. As described here, indoctrination into the LRA has unique aspects compared to many other armed groups. Realizing the far-reaching consequences of this, and how it affects individuals and the ways in which they interact with others and conceptualize social interactions, is critical for responding to the needs of child soldiers, and improving child health in conflict-affected regions. These results speak to the need for holistic programs targeted at trauma healing and reconciliation that involve both former abductees and community members.

## Footnotes

1 More recently, Lingala and Kiswahili are have also been spoken by the LRA, but Acholi remains the language spoken by the top leadership and many of the combatants.
